# O20 Approach to parotid swelling

**DOI:** 10.1093/rap/rkab067.019

**Published:** 2021-10-19

**Authors:** James Brighouse, Vinay Shivamurthy

**Affiliations:** 1 University Hospital Lewisham, London, United Kingdom; 2 Evelina London Children's Hospital, London, United Kingdom

## Abstract

**Case report - Introduction:**

The rarity of paediatric salivary gland disease and the lack of pathognomonic signs are likely to contribute to delay in diagnosis and make a structured approach to parotid swelling particularly important. Here we discuss a case of a 4-year-old girl with recurrent parotid swelling and some features to suggest multisystem involvement.

**Case report - Case description:**

A partially immunised, 4-year-old girl with a history of eczema and vitiligo presented with a 3-month history of intermittent painless pre-auricular swelling. She had 2 days of fever at onset but was otherwise afebrile. She reported intermittent joint pain without swelling. There was no cough, coryza, sore throat, dryness of the mouth or eyes, rash, systemic upset, and no other evidence of multisystem involvement on systems review. She had no unwell contacts and no family history of autoimmune disease. On examination she had bilateral pre-auricular swelling which was non-tender, not fluctuant, and had no overlying skin changes. No calculi were identified on bimanual palpation of the parotid ducts and no pus was visible at the opening. Respiratory, cardiovascular, abdominal, musculoskeletal, and skin examination were unremarkable.

Her bloods showed microcytic anaemia, raised ESR (peak 97mm/hr), CRP (peak 30mg/l), CK (peak 628 IU/l), and LDH (peak 512 IU/l), but normal ferritin and ACE. Serology showed ANA 1/10240, RNP Ab positive, negative myositis specific ENA, dsDNA, and rheumatoid factor, and normal complement. Infection screen, including TSpot, and screening for immunodeficiency were negative. Her urine dipstick was normal. Interferon signature was abnormal with very high levels. Parotid ultrasound scan showed heterogenous enlargement of all major salivary glands and lacrimal glands with reactive cervical lymph nodes. CT chest demonstrated basal ground-glass change and hilar, mediastinal, and axillary lymphadenopathy. Parotid biopsy was normal on two occasions, with no evidence of lymphoma or granulomatous disease.

At this stage she was treated for undifferentiated autoimmune connective tissue disease with steroids and mycophenolate mofetil. Due to the lack of improvement and persistently mildly elevated CK and LDH, MRI thighs was subsequently performed. This demonstrated mild myositis prompting a muscle biopsy which showed typical features of juvenile dermatomyositis. She was therefore commenced on intravenous immunoglobulin and subcutaneous methotrexate.

**Case report - Discussion:**

This is an unusual presentation of juvenile dermatomyositis with no typical clinical features of skin or muscle involvement, negative myositis ENA, and only mildly elevated CK, where parotitis was the main presenting feature.

Despite a dramatic reduction in mumps following routine immunisation, viral adenitis remains the most common cause of parotid swelling. The most prominent features are unilateral or bilateral parotid swelling with fever and headache and, unlike this case, typically resolve in 1—2 weeks. Bacterial adenitis may be suggested by erythema and purulent secretions and may be precipitated by preceding viral sialadenitis, dehydration, or damage by calculi. Obstruction and inflammation caused by calculi, sialolithiasis, are suggested by pain with meals and almost complete resolution in between. A single short episode of parotid swelling without multisystem involvement will most commonly be caused by one of the above and would not require extensive investigation. Management consists of one or more of: ensuring adequate hydration, warm compresses, analgesia, salivary gland massage, sialagogues, antibiotics, and safety netting advice.

More prolonged painful swelling despite sialagogues and antibiotics would warrant sialography to look for calculi, and prolonged painless swelling, particularly with the presence of any red flags, should prompt investigation for tumours or haematological malignancy.

Only in recurrent, treatment refractory episodes, or, in cases such as this, where a thorough history and examination suggest multisystem involvement, would more extensive investigation be warranted to look for inflammatory causes of parotitis.

**Case report - Key learning points:**

The differential diagnosis for parotid swelling is broad so a structured approach is essential. Extent of investigation should be guided by the history, particularly the clinical course and the evidence of multisystem involvement. It may be reasonable not to investigate at all in cases of isolated resolved or resolving parotid gland swelling without systemic upset and normal systems review. Where there are features to suggest multisystem disease, early referral to rheumatology for further investigation and treatment is needed.

